# Paper Matters: Technical Evaluation of Paper-Based Substrates for Enhanced Preconcentration of Biomolecules in Liquid Biopsy Diagnostics

**DOI:** 10.1021/acs.analchem.5c03749

**Published:** 2025-11-06

**Authors:** Panagiota M. Kalligosfyri, Antonella Miglione, Alessandra Glovi, Oğuzhan Aker, Valentina Arciuolo, Jussara Amato, Bruno Pagano, Concetta Di Natale, Sevinc Kurbanoglu, Ibrahim A. Darwish, Stefano Cinti

**Affiliations:** † Department of Pharmacy, 9307 University of Naples “Federico II”, 80131 Naples, Italy; ‡ Clinical and Translational Oncology Program, Scuola Superiore Meridionale (SSM, School of Advanced Studies), 9307 University of Naples “Federico II”, 80131 Naples, Italy; § Faculty of Pharmacy, 37504 Ankara University, 06560 Ankara, Turkey; ∥ Dipartimento di Ingegneria Chimica, dei Materiali e della Produzione Industriale, University of Naples Federico II, P.le Tecchio 80, I-80125 Naples, Italy; ⊥ Department of Pharmaceutical Chemistry College of Pharmacy, King Saud University, P.O. Box 2457, Riyadh 11451, Saudi Arabia; # Sbarro Institute for Cancer Research and Molecular Medicine, Center for Biotechnology, College of Science and Technology, Temple University, Philadelphia, Pennsylvania 19122, United States; ∇ Bioelectronics Task Force, University of Naples Federico II, Via Cinthia 21, 80126 Naples, Italy

## Abstract

Early detection of disease biomarkers remains a major challenge due to their low abundance in biological fluids. Preconcentration strategies can address this issue, but conventional approaches often require additional time, costly materials, or complex instrumentation. This work explores the use of paper-based substrates as a sustainable and cost-effective alternative for analyte preconcentration, highlighting how the choice of paper type critically influences sensitivity enhancement in analytical methods without compromising simplicity. Three cellulose-based papers (Whatman grade 1, Whatman grade 4, and a commercial laboratory filter paper) were systematically characterized and configured as 3D-origami-inspired, capillary-driven platforms. Their structural and fluidic properties were evaluated to determine how substrate selection affects analyte accumulation, transport, and detection efficiency. The devices were combined with three detection assays: a naked-eye colorimetric assay with smartphone integration, an electrochemical assay for miRNA, and a spectrophotometric Bradford assay for protein quantification. The paper platforms demonstrated rapid and efficient preconcentration across multiple biomarker classes. Up to a 20-fold enhancement was achieved for miRNA detection in human serum within 30 s, approximately 10-fold for short and double-stranded DNA in buffer, and about 2-fold for larger proteins. These results illustrate the importance of substrate selection and the versatility of paper-based preconcentration in handling analytes of different molecular sizes and assay formats. This study shows that cost-effective, customizable paper substrates can bridge the gap between high-performance biomolecular analysis and accessible diagnostics, enabling early disease detection and real-time monitoring in both laboratory and point-of-care settings.

## Graphical abstract

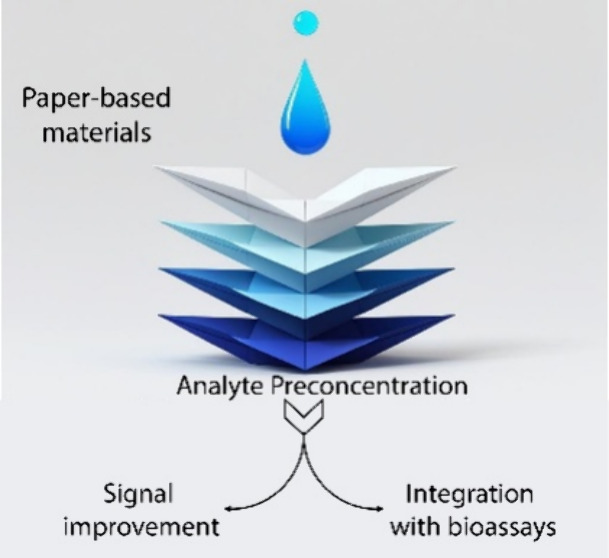

## Introduction

Biomolecules such as microRNAs (miRNAs), DNA, circulating tumor cells and proteins, including antibodies, have gained extensive attention as emerging biomarkers. These biomolecules are present in liquid biopsy samples collected from bodily biofluids such as blood, serum, saliva, and urine, offering valuable insights into disease detection and management.^1,2^ This interest has been further driven by the rise of liquid biopsy, particularly after 2010, during a period marked by increased research activity, technological development, and commercialization of diagnostic platforms.^3^ This minimally invasive approach allows real-time and continuous monitoring of disease, expanding the applications of biomarkers for early detection, prognosis, therapy selection, and treatment monitoring in personalized medicine.^2,3^

However, the detection of these biomolecules in biological samples is often complicated by their extremely low concentrations, particularly during the early stages of disease.^4–6^ To overcome this challenge, various preconcentration or enrichment techniques are employed to boost detection sensitivity prior to downstream analysis of proteins, nucleic acids, or microbial targets. Common strategies include ion exchange preconcentration columns,^7^ ultrafiltration, ultracentrifugation, immunomagnetic separation,^8^ fractionation by chromatography, differential solubility-based precipitation, and antibody-mediated immunoextraction.^5^ While these techniques are critical for enhancing detection limits, they frequently suffer from major limitations: they often involve expensive instruments, lengthy and complex protocols, and require well-equipped laboratory environments. Furthermore, most of these methods are not universally applicable, meaning that different biomolecules typically require different preconcentration systems. Nearly all these approaches also depend on external devices, such as pumps or isotachophoretic systems, for sample handling and delivery, which adds complexity and restricts their use in resource-limited or point-of-care (POC) settings.

To overcome the limitations of conventional preconcentration techniques, which often require bulky equipment, are time-consuming, and lack versatility, we developed an optimized self-driven, paper-based preconcentration device as a sustainable and accessible alternative. This device functions without the need for external instrumentation, enabling the rapid and efficient enrichment of nucleic acids, namely double-stranded (ds) DNA sequences and miRNAs, and proteins within just 1 min. Building on our previous work,^9^ we further investigated the use of various paper substrates. Paper’s inherent capillary action allows spontaneous fluid wicking and analyte transport without external pumps, making it an ideal substrate for low-cost, equipment-free preconcentration.^10^ In particular, we exploit cellulose-based filter papers (Whatman grade 1, grade 4, and commercial laboratory filter paper) due to their flexibility, reproducible structure, and ease of modification. Leveraging their inherent porosity and folding flexibility to optimize flow dynamics and enhance preconcentration performance, it was demonstrated that the choice of paper substrate significantly impacts sensitivity.^10–12^ By integrating these papers into origami-inspired 3D platforms, target molecules can accumulate efficiently in specific regions, enhancing detection across multiple assay formats while maintaining simplicity and affordability. In contrast to existing capillary-driven paper-based methods that depend on slow, repetitive drop-dry cycles or filtration steps, our approach enables multiple preconcentration cycles within the time frame of a single conventional step, significantly improving detection sensitivity without extending assay duration.

In order to demonstrate the efficacy and the promising application along with consolidated analytical procedures, three of the most known chromatographic/filter papers have been successfully integrated with colorimetric,^13^ electrochemical^14–17^ and spectrophotometric detection methods, demonstrating versatility across multiple analytical settings, both laboratory-based and decentralized. To provide significant insights, all the customized paper-based platforms have been custom-designed and characterized with electrochemical, morphological and flow-based strategies, also focusing on their retention and release capacity. Different biomarkers have been tested as the application: the optimized structures were applied in combination with an electrochemical sensor for miRNA detection, with a smartphone-powered colorimetric system for the detection of various sizes of dsDNA sequences and with the spectrophotometric Bradford assay for proteins detection. Depending on the molecule, paper properties, and device design, the 3D-origami platform achieves preconcentration factors of up to ∼20 within 30 s of rehydration, which occurs after the samples have first been dried for 15 min at room temperature. This work systematically evaluates diverse paper substrates and demonstrates a rapid, power-free approach that contrasts with isotachophoretic or pump-driven methods limited to PCR products.^18–20^ The optimized design enables single-step operation and seamless integration into multiple bioassays, minimizing the drying step to a single 15 min period, compared to other paper-based devices that require multiple, more extensive drying steps. Together, these features mark a clear advancement over our initial device, offering a versatile, efficient, and cost-effective strategy for biomolecular detection in laboratory and POC applications.

## Experimental Section

### Reagents and Instruments

Trehalose, potassium ferricyanide, DiSC_2_(5), Coomassie Blue dye, bovine serum albumin (BSA), serum and all other common reagents were obtained from Sigma-Aldrich (St. Louis, MO, USA). Synthetic nucleic acid sequences were prepared as described previously.^13^ Hypo-Opticlear Human serum was purchased from Sigma-Aldrich (St. Louis, MO,USA). The anti-miR21 DNA probe tagged with methylene blue (MB) (MB-tagged DNA probe) selective to miRNA-21, the target miRNA-21 sequence, (Table S1) were purchased from Metabion GmbH (Steinkirchen, Germany). For the preconcentration device, various paper-based substrates were used: (1) Whatman grade 1 filter paper (WF1) (Merck KGaA, Darmstadt, Germany) with a wicking speed of 150 s/100 mL, a thickness of 180 μm, and a pore size of 11 μm; (2) Whatman grade 4 filter paper (WF4) (Merck KGaA, Darmstadt, Germany), with a wicking speed of 37 s/100 mL, a thickness of 205 μm, and a pore size of 25 μm; (3) Commercial-grade laboratory filter paper (CFP) a thickness of 135 μm, intended for general use such as bench protection was obtained from MyCordenons (Milano, Italy). Light office paper, used for the colorimetric assay, was obtained from a local store. All paper substrates used in the preconcentration device assembly, as substates in the colorimetric assay, and as the platform for the screen-printed electrodes fabrication underwent wax printing, for 1 min at a temperature of 100 °C, to create hydrophobic barriers for controlled liquid handling. This process was performed using a ColorQube 8580 office printer (Xerox, USA), followed by heat treatment to ensure the formation of well-defined hydrophobic regions. A Xiaomi POCO X5 smartphone (Xiaomi Inc., Beijing, China) was used as the imaging device for assay detection. All captured images were processed using ImageJ, a freely available image analysis software (https://imagej.net/ij/index.html). Electrochemical measurements were carried out using an EmStat3 module single-channel portable potentiostat (PalmSens, Houten, The Netherlands) connected to a laptop. This setup enabled the electrochemical assessment of the paper-based substrates and precise electrochemical characterization of the preconcentrated miRNA samples. Spectrophotometric measurements for the Bradford assay were performed using a GENESYS 180 Ultraviolet–visible (UV–vis) Spectrophotometer (Thermo Fisher Scientific, Waltham, MA, USA).

### General Preconcentration Procedure

The self-driven, through capillary action, paper-based preconcentration device consisted of 10 layers of CFP substrate: 9 layers of 3 mm diameter discs and a 10th layer with a 9 mm diameter disc (Figure 1B). Previously performed optimization studies guided the design,^9^ as several layer configurations (3, 5, and 10 layers) were evaluated, with 10 layers providing the best balance between analyte diffusion time and preconcentration efficiency. The smaller 3 mm discs were used for sample application, ensuring efficient absorption of low-volume samples such as isolated DNA or miRNAs. Disc diameters for the final layer were also tested from 4 to 10 mm, with a 9 mm disc selected to serve as the preconcentration zone. The larger final disc acts as a self-driven pump to guide analyte movement and retains a higher quantity of analyte. This size also enables the integration of a screen-printed electrode (SPE) for electrochemical detection, creating a fully functional analytical platform (Figure S1).

**Figure 1:**
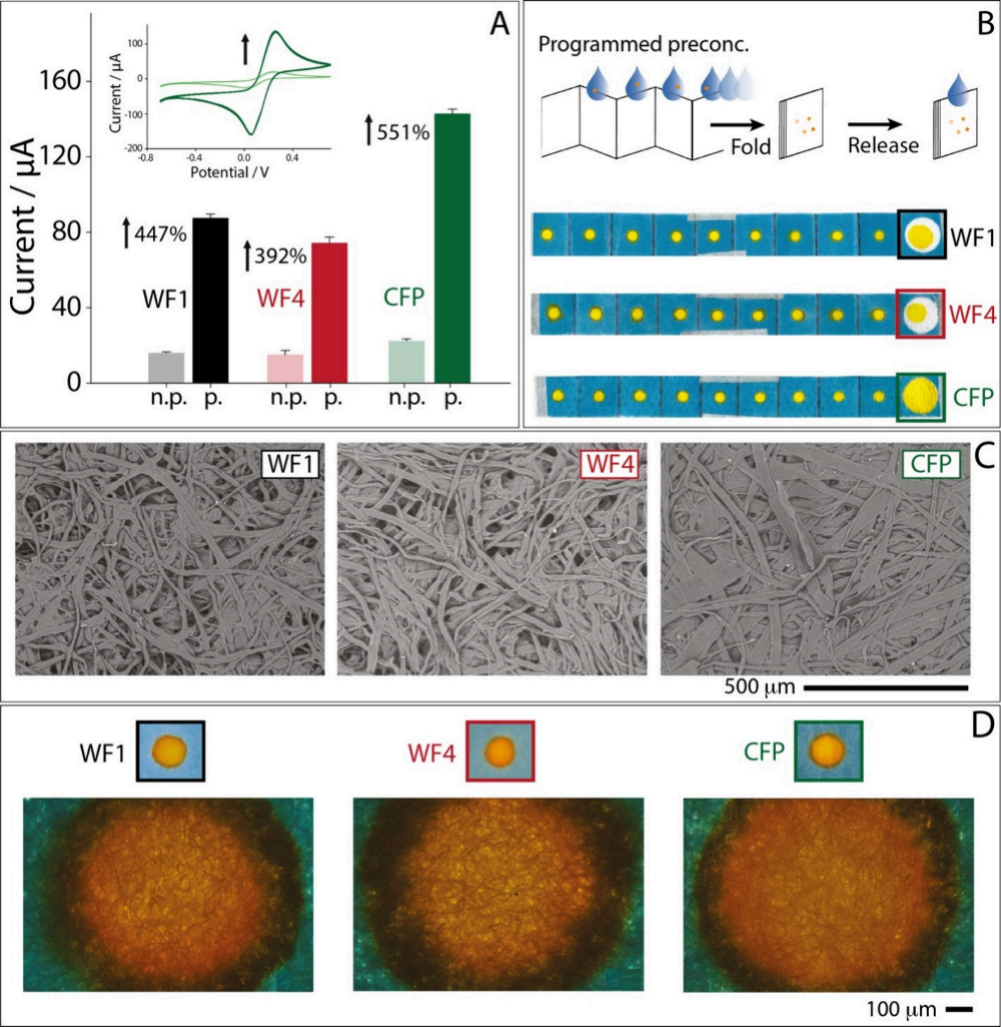
(A) Electrochemical evaluation of the preconcentration performance of the three paper-based substrates using cyclic voltammetry. Gray, light red, and light green bars represent the current response before preconcentration for the WFI, WF4, and CFP, respectively, and black, dark red, and dark green show values after preconcentration for the WF1, WF4, and CFP, respectively. Measurements were conducted at a scan rate of 0.05 V/s using 10 mM potassium ferricyanide as the model analyte, in six replicates. (B) Indicating dye migration experiments for 10 layers of WF1, WF4, and CFP preconcentration devices unfolded in 30 s. (C) SEM micrographs of the three paper-based substrates for morphological characterization at a 500 μm scale: (black) WF1, (red) WF4, and (green) CFP. (D) Microscopic analysis of the filter papers after the drying of the analyte. Color codes: black corresponds to WF1, red corresponds to WF4, and green corresponds to CFP. n.p: nonpreconcentrated, p: preconcentrated.

The device was integrated with three types of detection assays: a naked-eye colorimetric assay with smartphone integration for target quantification, an electrochemical assay for miRNA detection, a spectrophotometric method known as Bradford assay for protein quantification. For all three approaches, 10 μL of the desired analyte were applied and dried onto each of the 10 layers of the paper-based device. After drying at room temperature for 15 min, the device was folded into a vertical configuration, ensuring that each disc layer was in contact and positioned inside a 3D-printed case. The device relies on manual folding of multiple paper layers, which can introduce variability in layer alignment and contact uniformity. Therefore, misalignment may impair capillary flow continuity and reduce reproducibility. To address this, we have incorporated alignment aids (dashed vertical lines between every disc layer) that standardize layer placement and improve packing consistency. Once folded, 10 μL of water were added to the upper 3 mm disc layer. After 30 s, when the analyte had moved to the final 9 mm layer, the device was unfolded and left to dry at room temperature. For the smartphone-based colorimetric assay and Bradford assay the final disc, containing the preconcentrated analytes, was trimmed, while for the electrochemical assay the device was integrated with the SPE to directly quantify the preconcentrated target molecule (Figure 1B) The preconcentrated analyte was obtained by adding 50 μL of the reconstitution solution. The specific procedure for each detection assay is described further below.

### Colorimetric Assay for Preconcentrated Double-Stranded DNA Detection

The paper-based colorimetric assay was developed and optimized as reported previously.^13^ Calibration curves were constructed for both short (15 base pairs) and long (90 base pairs) double-stranded (ds)­DNA sequences containing 5 alternating A/T bases (Table S1). The short dsDNA sequences were prepared within a concentration range of 1–100 μM, while the ds-long sequences were in the range of 0.25–25 μM, both in 10 mM phosphate buffer (pH 7.4). To prepare the calibration curves, 8 μL of each sample was applied to a spot, designed on standard office paper, and allowed to dry at room temperature (RT). Once dried, 8 μL of a 50 μM DiSC_2_(5) dye solution, diluted in phosphate buffer, was added to each spot. The samples were incubated for 15 min at RT inside a 3D-printed dark chamber (Figure S2) to ensure consistent lighting conditions during image capture. Measurements were acquired using a smartphone camera.

The samples for the preconcentration step, using the paper-based based preconcentration device, were performed at three concentration levels: 1, 5, and 10 μM for short dsDNA sequences, and 0.25, 0.5, and 2.5 μM for the long dsDNA sequences, all in 10 mM phosphate buffer (pH 7.4). A 10 μL volume was applied in each paper disk and dried as described in the general preconcentration protocol. After dsDNA preconcentration and drying, the 10th 9 mm paper disc was trimmed, and the final layer was placed into an eppendorf vial. The preconcentrated analyte was extracted using 20 μL of 10 mM phosphate buffer (pH 7.4) and vortexed briefly. The resulting supernatant was carefully collected, dried onto a wax-treated light office paper substrate, and subsequently detected by adding 8 μL of DiSC_2_(5) solution. Following a 15 min incubation, the color intensity of the sequence-specific dye was measured using a smartphone camera, as previously reported.^13^ The images were captured in the designated 3D-printed dark chamber under controlled lighting conditions, and were further analyzed using ImageJ, an open-access software. The results were interpreted as the percentage (%) of signal difference between the blank sample (dried PB buffer) and the sample containing the nucleic acid target, where the signal corresponds to the color intensity of the dye measured as pixel intensity in the images. The percentage signal difference was then correlated with the nucleic acid concentration.

### Electrochemical Assay for Preconcentrated microRNA Detection

The screen-printed electrochemical sensor for miRNA detection was fabricated as described previously.^14–17^ Briefly, office paper was employed as the SPEs substrate. For optimal biosensor performance, the working electrode was modified by drop-casting 4 μL of gold nanoparticles (AuNPs), enabling large-scale fabrication while maintaining efficiency. AuNPs (monodispersed suspension with an average diameter of 196.2 ± 20.7 nm^21^) provided a stable platform for immobilizing the thiol-functionalized DNA probe (anti-miRNA-21), which was reduced with 10 mM tris­(2-carboxyethyl)­phosphine (TCEP) for 1 h at room temperature (RT) to activate covalent binding via Au–S bonds. The probe solution was then diluted and immobilized onto the AuNP-modified electrode by incubating 20 μL of the probe for 1 h at RT in a humid chamber. To passivate unoccupied electrode areas, 2 mM 6-mercapto-1-hexanol was applied for 1.5 h, followed by thorough washing with distilled water. The detection strategy utilized a “signal-off” approach, where hybridization-induced conformational changes affected electron transfer. In the absence of target miRNA-21, the probe retained its hairpin structure, allowing efficient electron transfer. Upon hybridization, structural rearrangement hindered electron transfer, enabling miRNA-21 detection via electrochemical signal changes.

The preconcentration process involved applying a 10 μL aliquot of the sample onto each layer of the device, followed by air drying at room temperature. Once fully dried, the origami structure was systematically folded, allowing the accumulated target analyte to concentrate within the final layer. To recover the preconcentrated miRNA, 10 μL of buffer solution was introduced onto the terminal layer, facilitating rehydration. This final layer, where the screen-printed electrode was printed, was then dried, carefully excised, and the preconcentrated sample was reconstituted in 50 μL of PBS buffer. The obtained 50 μL sample was subsequently subjected to electrochemical analysis utilizing square wave voltammetry (SWV): *t*_equilibration_ = 5 s, *E*_start_ = 0.0 V, *E*_end_ = – 0.5 V, *E*_step_ = 0.001 V, amplitude = 0.01 V, frequency = 50.0 Hz. The results were interpreted as percentage signal change that was determined by comparing the current recorded without miRNA (blank sample) to the current measured after target miRNA addition, expressing the relative decrease as a percentage of the initial signal. This calculation quantifies the extent of signal variation induced by target binding. This electrochemical approach enabled precise quantification of the target miRNA, enhancing detection sensitivity through the effective preconcentration of the analyte within the device.

### Bradford Assay for Protein Detection

The Bradford assay was employed to quantify protein concentrations using a Coomassie Brilliant Blue G-250 dye-based colorimetric method.^22,23^ To generate a calibration curve, a series of BSA standards was prepared by diluting a 1 mg/mL BSA stock solution with 150 mM NaCl to achieve the desired concentrations. The standard concentrations used were: 0.01, 0.025, 0.05, 0.1, 0.2 and mg/mL. A blank control containing only 150 mM NaCl was also included for baseline correction.

The Coomassie Blue G-250 dye solution was freshly prepared by dissolving 0.0025 g of Coomassie Blue in 5 mL of absolute ethanol. To this solution, 10 mL of 85% phosphoric acid (H_3_PO_4_) was added, followed by dilution with distilled water to a final volume of 100 mL.^23^ This reagent forms a blue dye-protein complex whose intensity is proportional to protein concentration.^22^

For each calibration standard, 1 mL of the sample was mixed with 50 μL of the prepared Coomassie dye solution and incubated for 5 min at room temperature. Absorbance was then measured at 595 nm, the optimal wavelength for detecting the dye-protein complex. The absorbance values were used to construct a calibration curve, which was later used to quantify protein in unknown samples.

In the preconcentration step, BSA solutions at concentrations of 0.01, 0.025, and 0.05 mg/mL were tested using a paper-based preconcentration device. A 10 μL aliquot of each BSA solution was applied to individual discs on the device and allowed to dry at room temperature. After drying, 10 μL of working buffer was applied to the top layer to rehydrate and mobilize the preconcentrated protein. The bottom layer of the device, containing the concentrated BSA, was carefully cut and recovered. The preconcentrated protein was then reconstituted in 50 μL of buffer and mixed with 1 mL of Bradford reagent for quantification. The absorbance was measured at 595 nm to determine the final protein concentration.

## Results and Discussion

### Preconcentration Mechanism

The device consists of 10 paper layers, with the top nine designed as 3 mm diameter discs and the tenth, bottom layer enlarged to 9 mm to act as a “pump,” facilitating the absorption and vertical movement of the analyte toward this collection zone. Hydrophobic barriers created by wax printing precisely define circular fluidic paths, preventing lateral leakage and ensuring controlled, directional liquid transport. Each of the nine smaller discs is preloaded with the analyte, stabilized with trehalose to promote homogeneous drying and rehydration, and subsequently stacked in an origami configuration to maintain direct vertical contact between layers. Upon addition of water to the top disc, the dried analyte is rehydrated and mobilized, allowing it to be carried through the underlying layers by capillary action, sequentially dissolving and transferring the analyte originally present in each subsequent layer. The liquid spontaneously wicks downward through the porous cellulose network via capillary action, a process mediated by surface tension and the adhesive interactions between water molecules and hydrophilic paper fibers. Much like a paper towel absorbing a spill without external force, this vertical capillary flow continuously drives the solvent front through the stacked layers, efficiently mobilizing analyte from each disc and progressively concentrating it into the larger bottom layer. This stepwise, vertically directed transport maximizes preconcentration by ensuring that analyte from multiple layers converges in a single, defined collection zone. The enlarged bottom disc (10th) localizes the enriched analyte after a short incubation, providing a simple, low-cost, and pump-free platform for highly efficient preconcentration and downstream analysis (Figure S3).

### Preconcentration Evaluation of the Paper-Based Substrates

To evaluate the preconcentration efficiency of candidate paper-based substrates, we employed an electrochemical approach using potassium ferricyanide as a model analyte. The optimized 10-layer origami configuration, as previously described,^9^ was used to assess the performance of three different substrates: WF1, WF4, and CFP. For the preconcentration evaluation, 10 μL of 10 mM potassium ferricyanide solution was applied to the unfolded paper-based preconcentration device and allowed to dry. After folding the origami device, the preconcentrated analyte was eluted from the final (10th) layer by adding 10 μL of water. While increasing the number of layers can enhance analyte enrichment,^9^ the layer number must be carefully matched to the sample volume to ensure proper drying and analyte release. For instance, 20 layers with 20 μL drops produced high preconcentration factors, but such configurations may be less suitable for low-volume samples, highlighting the need to optimize layer design for different sample sizes.

Cyclic voltammetry (CV) was performed to determine the analyte concentration before and after preconcentration. Measurements were conducted at a scan rate of 0.05 V/s. As shown in Figure 1A, the gray, light red, and light green bars represent the current responses before analyte preconcentration for WF1, WF4, and CFP, respectively. In contrast, the black, dark red, and dark green bars depict the current values after preconcentration for WF1, WF4, and CFP. These current responses were obtained from CV measurements.

CFP demonstrated the highest preconcentration efficiency, exhibiting a 551% increase in signal intensity, outperforming both WF1 (447%) and WF4 (392%) substrates. The detailed CV curves obtained before and after preconcentration for each paper type are presented in the Supporting Information (SI) (Figure S3 for WF1, Figure S4 for WF4, and Figure S5 for CFP). The superior performance of the commercial filter paper (CFP) observed in electrochemical measurements aligns with the dye migration experiments (Figure 1B). A 10 μL solution of yellow food dye was deposited on the top layer of the folded preconcentration device to initiate analyte migration. Devices consisting of 5, 10, and 20 layers were investigated for all paper-based substrates namely WF1 (Figure S7), WF4 (Figure S8), and CFP (Figure S9). In the 20-layer configuration, the effect of wicking and migration speed was even more pronounced in the CFP substrate. The devices were unfolded at specific time intervals namely 20 s, 30 s, 40 s, 1 min, and 2 min, to monitor dye movement. These experiments revealed that the CFP supported the fastest flow. Notably, at just 20 s, it was the only substrate capable of transporting the analyte to the 10th (final) layer and within 40 s to the 20th (final) layer, where preconcentration occurs. This confirms that CFP exhibited the fastest wicking speed, followed by WF1 and then WF4.

Additionally, a rehydration experiment was conducted to evaluate the recovery efficiency of each paper substrate (Figure S10). In this test, 10 μL of yellow food dye was first dried on the surface of each substrate. Subsequently, a 10 μL drop of water was applied directly onto the dried dye, allowing passive rehydration to occur without any mechanical agitation. The water droplet was left in contact with the substrate for various time intervals: 30 s, 1, 2, 5, and 10 min. The results for the reconstitution efficiency were evaluated according to the absorbance intensities of the dried dye and the dye solution. After 10 min, WF1 recovered approximately 75% of the original dye signal, WF4 recovered about 70%, and CFP demonstrated the highest recovery at 96%. These percentages indicate that CFP facilitates nearly complete reconstitution of dried dye, while WF1 and WF4 exhibit comparatively lower recovery, likely due to differences in fiber structure and water interaction dynamics. This result indicates better interaction between the water droplet and the fiber structure of the CFP substrate, leading to a higher rehydration efficiency.

Following rehydration, the recovered dye from each substrate was diluted to a final volume of 500 μL and analyzed using UV–vis spectrophotometry. The results confirmed superior analyte recovery for the CFP substrate. This experiment was carried out for all paper substrates under investigation: WF1 (Figure S11A), WF4 (Figure S11B), and CFP (Figure S11C). All corresponding absorbance spectra for each paper type at the various rehydration time points are presented in the SI.

To further investigate the analyte migration mechanism within the CFP-based origami device, the substrate with superior performance, potassium ferricyanide was employed as a model compound. Calibration curve was obtained from dried and reconstituted potassium ferricyanide solutions (1–100 mM) prepared in 0.1 M KCl (Figure S12A) as discussed in detail in the SI. The distribution of analyte across the layers after the preconcentration step was quantified (Figure S12B, Table S2). The results revealed a clear gradient of analyte migration through the device, with progressive accumulation toward the final layers. Layers 1–6 contained only minor amounts of analyte (1.48–3.68% of total mass), whereas layers 7–9 exhibited increased retention (5.22–6.32% of total mass). The majority of the analyte, 214.08 μg (65.02% of the total), was recovered in the 10th layer, demonstrating the directional transport and preconcentration efficiency of the device. The total analyte recovery was 320.02 μg from an initial 329.24 μg applied, corresponding to an overall recovery of 97.2% and a minimal total loss of 2.8%. These findings confirm that the origami configuration effectively promotes analyte migration and accumulation in the final layer, while maintaining good mass recovery, thereby validating its suitability for preconcentration-based analytical applications.

### Physical Characterization of the Paper-Based Substrates

To identify the optimal substrate for analyte drying and preconcentration efficiency, three different paper-based materials WF1, WF4 and CFP were systematically evaluated. Scanning Electron Microscopy (SEM) was employed to characterize the microstructural features of each substrate, and fiber morphology was quantitatively analyzed using ImageJ software. As shown in Figure 1C, the three substrates exhibit distinct microstructural properties in terms of porosity, fiber diameter, and pore distribution.

SEM imaging revealed that CFP possesses significantly wider fibers compared to WF1 and WF4. Although CFP substrate appears denser under SEM characterization with less visibly distinct pores exhibited higher permeability and faster fluid flow. This behavior can be attributed to the formation of larger interstitial voids between the broader fibers, which facilitate more efficient fluid migration.^11,24,25^

These structural observations align with the electrochemical measurement of analyte transfer efficiency. CFP demonstrated the highest transfer rate and preconcentration efficiency (fiber width: 23.20 ± 3.28 μm), significantly outperforming WF1 (fiber width: 13.69 ± 4.61 μm) and WF4 (fiber width: 14.39 ± 6.37 μm). Additionally, CFP exhibited a lower standard deviation, indicating greater consistency and reproducibility in fluid handling. In contrast, Whatman filter papers displayed finer, more uniformly packed fibers, resulting in smaller pore sizes and slower analyte migration. These findings emphasize the critical influence of fiber diameter and pore dimension on fluid transport properties in the paper-based preconcentration origami device. In particular, the wider fibers and larger void spaces in CFP enabled rapid analyte preconcentration without the need for external equipment, enhancing the performance of the origami-based device and is in accordance with the electrochemical evaluation of this specific paper-based substrate.

Microscopic visualization of the dried analyte further confirmed these results. The coffee-ring effect^26^ was visibly reduced by approximately 2-fold in the CFP substrate compared to WF1 and WF4. In CFP, dye deposits appeared homogeneous and well-distributed, as shown by color intensity in Figure 1D. The coffee-ring effect occurs when particles in a drying droplet migrate toward the edges, forming a ring-like deposit and leaving the center of the paper substrate emptier, which can reduce the reproducibility and sensitivity of preconcentration assays.^26^ In our previous study,^9^ the addition of 1% trehalose promoted homogeneous drying, suppressed the coffee-ring effect, and resulted in up to a 2-fold increase in signal intensity. The addition of sugar reduces the coffee-ring effect by slowing down the outward capillary flow in the droplet. As the droplet dries, sugar precipitates near the edges, which pins and then stabilizes the contact line while forming a solid-like boundary. This mechanism suppresses the usual particle migration to the edges, leading to a more uniform deposition.^26^ To quantify the coffee-ring effect, we performed image analysis using ImageJ. CFP exhibited a 20% larger diameter than WF1 and 16% larger than WF4, and intensity analysis showed that the total signal on CFP was approximately 8% higher than on the other two substrates. Considering CFP’s larger effective area, this indicates that it retained more analyte overall. Finally, CFP facilitates uniform analyte deposition, faster flow rates, enhanced drying/reconstitution, and higher electrochemical signals, making it an optimal substrate for capillary-driven preconcentration. In summary, despite lacking visible porosity under SEM, the CFP substrate exhibited superior overall performance in this multi layered origami device. Overall, the combination of substrate morphology and trehalose enabled uniform, reproducible, and sensitive preconcentration. To better contextualize the performance of the different substrates, the key physical and structural properties of the tested papers (WF1, WF4, and CFP) are summarized in Table S3.

Based on these results, CFP was selected as the preferred substrate for integration with both colorimetric, electrochemical assays and spectrophotometric assays targeting dsDNA, miRNA and protein analytes, respectively.

### Colorimetric Assay for Preconcentrated dsDNA Detection

For both short and long dsDNA sequences containing the TATA motif, i.e. five alternating A/T base pairs, calibration curves were constructed in buffer solution using nonpreconcentrated samples. The short dsDNA sequences (15 base pairs) were prepared in the concentration range of 1–100 μM, while the long sequences (90 base pairs) ranged from 0.25–25 μM. Detection was carried out using a smartphone-based detector, with 50 μM of a sequence-specific dye added as previously described.

For the short dsDNA sequences, the signal responses of preconcentrated samples were compared to those of the nonpreconcentrated samples (Figure 2A). The developed paper-based preconcentration device demonstrated a notable enhancement in signal intensity for the long sequences, with increases of up to 122% observed at three concentration levels (Figure 2B). These improvements were calculated using the respective calibration equations.

**Figure 2:**
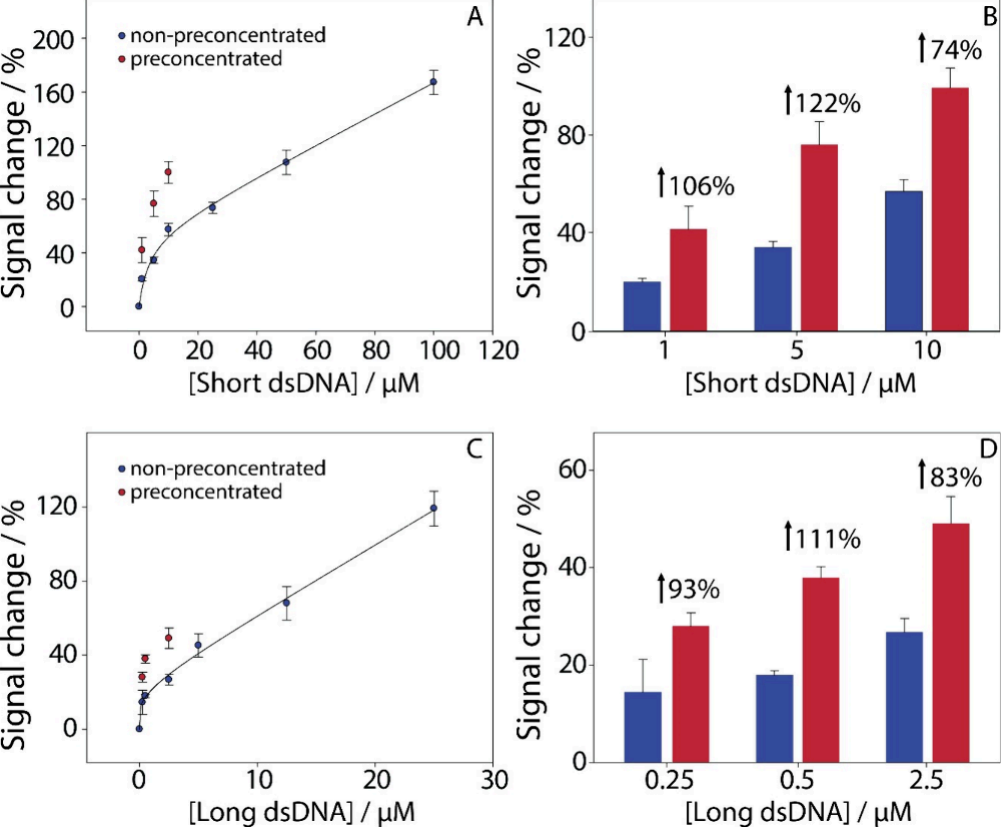
(A) Calibration curve obtained for the nonpreconcentrated (blue circles) short dsDNA sequences within the range of 0–100 μM and the corresponding preconcentrated (red circles) of the three lower dsDNA concentrations 1, 5, and 10 μM. (B) %signal improvement of the colorimetric assay by using the paper-based origami device. In blue, the nonpreconcentrated values and in red color, the preconcentrated values. (C) Calibration curve obtained for the nonpreconcentrated (blue circles) long dsDNA sequences within the range of 0–25 μM and the corresponding preconcentrated (red circles) of the three lower dsDNA concentrations 1, 5, and 10 μM. (D) %signal improvement of the colorimetric assay by using the paper-based origami device. In blue, the nonpreconcentrated values and in red color, the preconcentrated values. All experiments were performed in quadruplicate.

The calibration equation for the short dsDNA sequences was determined tobe *y* = 7.58 + 5.13*x* (*R* ^2^ = 0.98) with a limit of detection (LOD) of 23.52 nM. The preconcentration process resulted in substantial signal enhancements, with an increase in concentration of up to 7-fold at 1 μM, 2.7-fold at 5 μM, and 2-fold at 10 μM. Similarly, for the long dsDNA sequences (0–25 μM) a calibration curve was prepared (Figure 2C) for the nonpreconcentrated targets and the effect of preconcentration was quantified. A significant signal enhancement up to 111% was observed across all samples (Figure 2D). Based on the calibration curve: *y* = 15.93 + 4.24*x* (*R* ^2^ = 0.99) with a LOD of 5.15 nM, the signal enhancements corresponding to concentrations of 0.25, 0.5, and 2.5 μM were calculated to be approximately 8-fold, 8-fold, and ca. 3-fold, respectively, underscoring the high sensitivity and efficiency of the paper-based device. The achieved linear range and LOD for the longer dsDNA sequences were lower compared to the shorter TATA sequences. This outcome is expected because longer amplicons provide greater structural complexity, which can reduce the efficiency of dye intercalation and fluorescence response. In particular, the presence of nonalternating AT regions in the longer sequences creates additional binding sites where the dye may interact nonspecifically or with reduced affinity. However, it is important to note that when the same sequences lacking the TATA motif was previously tested,^13^ no significant interfering effects were observed, suggesting that the reduced performance is specifically linked to sequence composition rather than general nonspecific interactions. Finally, the repeatability of the assay (*n* = 3) was expressed as the percentage relative standard deviation (%RSD). All %RSD values obtained were between 10 and 13% demonstrating the high repeatability of the colorimetric assay.

These results highlight the potential of the developed system as a powerful and accessible tool for amplifying detection signals, particularly in low-concentration analyte detection. Its simplicity and portability make it especially suitable for POC and field applications.

### Electrochemical Assay for microRNA Detection

The robust miRNA electrochemical detection assay previously reported^17^ was utilized as a proof of concept of our device. To overcome the challenges associated with detecting trace concentrations of miRNA in complex liquid biopsy samples, our proposed paper-based preconcentration device was employed. Before the preconcentration step, the paper-based SPE was assessed by performing calibration graphs in spiked human serum (Figure 3A) with a concentration range of 0.1–1000 nM of miRNA target. The 4-Hill parameter model was utilized, and the following equation was obtained: *y* = (3.8 + 65.7 × *x* ^0.8^)/(76.8^0.8^ + *x* ^0.8^) (*R* ^2^ = 0.99) with a LOD of 1.2 nM. This equation was then used to quantify the enhanced signal from the preconcentrated samples.

**Figure 3:**
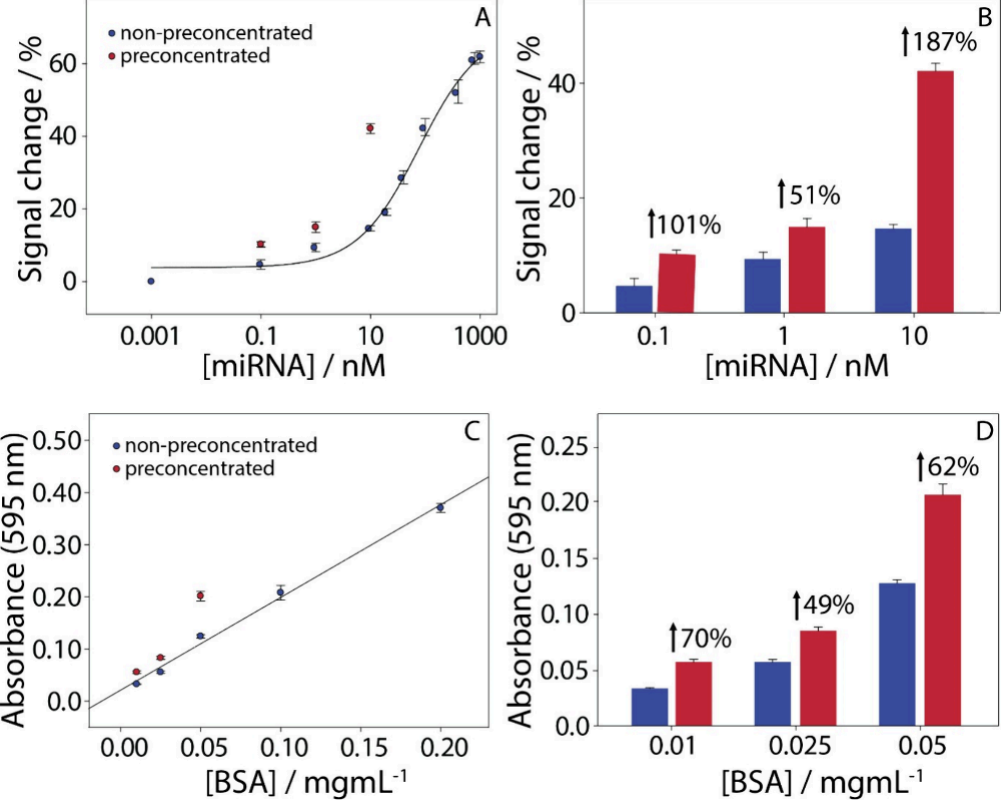
(A) Calibration curve obtained in spiked serum for the nonpreconcentrated (blue circles) miRNA target within the range of 0–1000 nM and the corresponding preconcentrated (red circles) of the three lower miRNA concentrations 0.1, 1, and 10 nM. (B) %signal improvement of the colorimetric assay by using the paper-based origami device. In blue, the nonpreconcentrated values and in red color, the preconcentrated values. (C) Calibration curve obtained in working solution for the nonpreconcentrated (blue circles) BSA protein molecules within the range of 0–0.2 mg/mL and the corresponding preconcentrated (red circles) of the three lower BSA concentrations 0.01, 0.025, and 0.05 mg/mL. (D) %signal improvement of the Bradford assay by using the paper-based origami device. In blue, the nonpreconcentrated values and in red color, the preconcentrated values. All experiments were performed in triplicate.

After the analytical assessment of the electrochemical biosensor, the preconcentration paper-based device was applied to untreated, spiked human serum samples to evaluate its effectiveness under biologically relevant conditions. A signal enhancement of up to 187% was observed (Figure 3B). Notably, up to 18-fold preconcentration efficiency was consistently achieved across all tested concentration levels, 0.1, 1, and 10 nM. A good repeatability, i.e. %RSD, of ca. 8% was obtained across measurements of the serum samples (*n* = 3).

These results demonstrate the practical applicability of the paper-based preconcentration device for real sample analysis. Moreover, the use of paper substrates appears to mitigate matrix effects commonly associated with complex biological samples, eliminating the need for additional pretreatment steps.^11,27,28^ The integration of preconcentrated values into the calibration curve highlights the substantial impact of miRNA preconcentration on biosensor performance. This approach boosted the electrochemical signal of the target miRNA by nearly 10-fold. Remarkably, a preconcentrated sample at 0.1 nM produced a signal comparable to that of a nonpreconcentrated 1.6 nM sample, demonstrating its efficiency in enhancing detection sensitivity, even in complex biological matrices like serum. Our preconcentration method increases the effective concentration of miRNA 16-fold. This enables detection of samples as if they were concentrated 16 times higher, equivalent to achieving sensitivity even down to 6 pM. Such performance reaches well below physiologically relevant levels,^29,30^ supporting reliable detection of low-abundance miRNA for early cancer diagnosis and monitoring.

### Bradford Assay for Protein Detection

To further demonstrate the potential of the optimized origami device, we applied it to protein samples using the well-established Bradford assay for protein quantification. A calibration curve (Figure 3C) was generated in solution using BSA protein standards ranging from 0 to 0.2 mg/mL alongside measurements of preconcentrated samples at the three lowest concentrations: 0.01, 0.025, and 0.05 mg/mL). The resulting calibration equation was *y* = 0.019 + 1.78 × *x* with a high linear correlation (*R* ^2^ = 0.99) and a calculated LOD of 0.052 mg/mL. Preconcentration with the origami device was applied specifically to the lowest BSA concentrations (0.01, 0.025, and 0.05 mg/mL). The signal enhancement achieved for these samples was up to 2-fold compared to the nonpreconcentrated measurements. Figure 3D shows the percentage signal improvement, with nonpreconcentrated values in blue and preconcentrated values in red.

The repeatability of the integrated assay was assessed by calculating the %RSD, which ranged between 1.7 and 6.7% (*n* = 3), indicating high reproducibility of the spectrophotometric assay coupled with the origami-based preconcentration platform.

These results underscore the versatility of the device in handling analytes of varying nature and molecular sizes and confirm its compatibility with diverse bioassays. This adaptability is further highlighted in Table 1 that summarizes diverse paper-based preconcentration methods reported in the literature, showcasing variability in analytes, sample matrices, and detection techniques. Electrokinetic trapping on filter paper, for instance, achieves high preconcentration factors (up to 170×) in short durations (80 s).^18^ However, this approach relies on external power, limiting its applicability for POC diagnostics, and has been validated primarily in buffer systems rather than complex biological matrices. Such methods often face practical constraints due to their dependence on additional instrumentation, which compromises portability and user-friendliness.

**Table 1:** Various Preconcentration Approaches Reported Utilizing External or Self Driving Forces^a^

paper-substrate	precon. approach	vol. of sample (μL)	analyte - matrix	assay	precon. factor	precon. time (s)	ref
external equipment							
filter paper	electrokinetic trapping	200	ssDNA buffer	colorimetric	170	80	^18^
self-driven via capillary forces							
filter paper	drop cast	10	copper - serum	electrochemical	5	N/A	^31^
filter paper	drop cast	10	miRNA-serum	electrochemical	10	N/A	^32^
filter paper	programmable-capillary action	100	glucose - sweat	electrochemical	3	30	^9^
common bench filter paper	origami-based – capillary action	80	dsDNA (15 and 90 base pairs)	colorimetric	8.6	30	this work
common bench filter paper	origami-based – capillary action	100	miRNA serum	electrochemical	18	30	this work
common bench filter paper	origami-based – capillary action	100	protein	spectrophotometric	2	30	this work

a Precon: preconcentration, ssDNA: single stranded DNA, and dsDNA: double-stranded DNA.

In contrast, self-driven capillary-force systems including drop-casting and programmable capillary action provide simpler, equipment-free platforms.^9,31,32^ These methods achieve modest preconcentration factors (3- to 18-fold), depending on the analyte and detection assay principle.

To sum up, preconcentration methods such as electrokinetic trapping require external electrical power, precise microfabrication, and often operate on very small sample volumes, limiting their ease of use and scalability. Drop-casting approaches lack fluid control and yield lower enrichment factors. In contrast, our origami-based, capillary-driven device is self-powered by intrinsic paper properties, enabling equipment-free operation, higher sample volumes, and efficient analyte concentration with minimal user steps. This simplicity and scalability represent clear advantages for POC applications.

Notably, our work advances this field by achieving an 18-fold preconcentration efficiency for miRNA detection in serum within 30 s of reconstitution, after the analyte has been dried on standard filter paper. To our knowledge, this platform also demonstrates a 9-fold enhancement for dsDNA in buffer, a capability not previously reported for nucleic acids for self-driven systems. Finally, for larger molecules like proteins, a 2-fold preconcentration efficiency was observed. The final preconcentration efficiency, based on our results, is primarily determined by intrinsic molecular properties such as size, hydrophilicity, and diffusion coefficients. Smaller molecules, such as miRNAs, are enriched more effectively than larger DNA or proteins, and the detection assay used also influences the observed efficiency. Although both the analytes^33–35^ and cellulose^36^ carry negative charges under the assay conditions, resulting in nonfavorable electrostatic interactions, these do not dominate analyte retention or migration, indicating that charge alone does not account for the differences in preconcentration across analytes. Spectrophotometric methods may exhibit signal saturation, limiting apparent enrichment, while electrochemical assays can provide higher or more linear responses. These variations reflect the combined effects of molecular properties, paper characteristics, and detection modality, emphasizing the need for further optimization to improve integration across diverse biomolecular assays.

This performance matches or exceeds that of existing equipment-free strategies, while maintaining simplicity, cost-effectiveness, and compatibility with POC and broader application settings. The platform’s multifunctionality, including efficient miRNA preconcentration from biological fluids, robust colorimetric detection of double-stranded DNA, and effective protein preconcentration coupled with Bradford assay quantification, demonstrates its broad applicability. These findings underscore the potential of paper-based microfluidics to enable rapid, high-yield preconcentration without the need for specialized equipment, particularly in diagnostic settings involving complex biological samples.

## Conclusions

Paper-based substrates and their on-demand configurations represent a valuable tool for making existing analytical procedures more sensitive, by acting as sustainable and frugal devices for preconcentration. However, paper-based is too general as a concept, and it should be technically evaluated which is the porous paper providing the most relevant enhancement of the analytical performance. As highlighted in the manuscript, the inherent affordability of paper-based substrates contributes to cost-effectiveness by minimizing the need for expensive materials and reducing both reagent and sample consumption, by facilitating efficient analyte transport and signal enhancement.

The frugal idea of this technical note is based on the ability of paper to be customizable within 3D-structures and to enhance the sensitivity of multiple assay formats, including both colorimetric, optical and electrochemical methods. The satisfactory application of this universal concept has been evaluated toward the analysis of three relevant biomarkers in liquid biopsy, namely double-stranded DNA, miRNA and proteins (Table S4).

Utilizing an origami-inspired, capillary-driven design, the platform achieves rapid and efficient target accumulation, delivering up to ca. 20-fold enhancement in miRNA detection from human serum in just 30 s of rehydration. Additionally, the device achieved up to approximately 10-fold preconcentration efficiency for both short and double-stranded DNA in buffer, an impressive result for a self-driven, paper-based system. For larger protein molecules, a 2-fold preconcentration efficiency was observed. These outcomes highlight the device’s versatility in handling analytes of different molecular sizes and functionality, as well as its compatibility with a range of bioassays. Moreover, the consistent performance across various targets underscores the device’s reliability, even when applied to complex biological samples.

This work emphasizes the transformative potential of paper-based preconcentration technologies in molecular diagnostics and liquid biopsy applications, paving the way for early disease detection and real-time therapeutic monitoring, particularly in resource-limited environments. Future directions for the proposed preconcentration device include expanding its application to other analytes such as extracellular vesicles, and antibodies, as well as testing with a wider variety of biological samples beyond serum and sweat, including blood, urine, and even real PCR-amplified double-stranded DNA products. Additionally, its programmable and modular nature makes it compatible with various detection techniques, further enhancing its adaptability and potential in bioanalytical applications.

## Supplementary Material



## Notes

Table of DNA and miRNA sequences; colorimetric assay configuration; electrochemical characterization of the paper-based substrates; dye migration studies; analyte mitigation studies; table of mitigation studies; table of key properties of the paper-based substrates; and table of preconcentration efficiency across targets and assays (PDF)
