# Supplementary material for: Paper Matters: Technical Evaluation of Paper-Based Substrates for Enhanced Preconcentration of Biomolecules in Liquid Biopsy Diagnostics
Source: Anal Chem. 2025 Nov 6;97(45):24936–45. doi: 10.1021/acs.analchem.5c03749 (PMC12631723; doi:10.1021/acs.analchem.5c03749)
Supplement: Supplementary file 1 [file ac5c03749_si_001.pdf]

Supporting Information

Paper matters: technical evaluation of paper-based substrates for enhanced preconcentration of biomolecules in liquid biopsy diagnostics

Panagiota M. Kalligosfyri[1*], Antonella Miglione[1], Alessandra Glovi[1,2], Oğuzhan Aker[3], Valentina Arciuolo[1], Jussara Amato[1], Bruno Pagano[1], Concetta Di Natale[4], Sevinc Kurbanoglu[3], Ibrahim A. Darwish[5] and Stefano Cinti[1,6,7*]

[1]Department of Pharmacy, University of Naples "Federico II", 80131 Naples, Italy
[2]Clinical and Translational Oncology Program, Scuola Superiore Meridionale (SSM, School of Advanced Studies), University of Naples "Federico II", 80131 Naples, Italy
[3]Faculty of Pharmacy, Ankara University, 06560, Ankara, Turkey
[4]University of Naples Federico II, Dipartimento di Ingegneria Chimica, dei Materiali e della Produzione Industriale, P.le Tecchio 80, I-80125 Naples, Italy
[5]Department of Pharmaceutical Chemistry College of Pharmacy King Saud University P.O. Box 2457, Riyadh 11451, Saudi Arabia
[6]Sbarro Institute for Cancer Research and Molecular Medicine, Center for Biotechnology, College of Science and Technology, Temple University, Philadelphia, PA 19122, USA
[7]Bioelectronics Task Force at University of Naples Federico II, Via Cinthia 21, 80126 Naples, Italy
*Email: panagiota.kalligosfyri@unina.it, stefano.cinti@unina.it

Table of content

Table S1. DNA and miRNA sequences Page S2
Integration of the paper-based preconcentration device with a screen-printed electrochemical sensor. Page S2
Figure S1. Integrated paper-based preconcentration device with a screen-printed sensor. Page S3
Figure S2: Assay configuration of the colorimetric nucleic acid detection. Page S3
Figure S3. Preconcentration mechanism**.** Page S4
Preconcentration evaluation of the paper-based substrates. Page S4
Figure S4-S6. Electrochemical evaluation of the preconcentration efficiency. Page S5-S6
Dye migration experiments. Page S6
Figure S7-S9. Dye migration experiments for the three paper-substrates. Page S7-S9
Analyte recover efficiency. Page S9
Figure S10: Visual comparison of the three paper-based substrates. Page S10
Figure S11: Rehydration experiment for all paper-based substrates. Page S11
Analyte mass transfer studies. Page S11
Figure S12. Analyte mass transfer quantification. Page S12
Table S2. Analyte mass transfer quantification and total mass percentages. Page S12
Table S3: Key properties and physical characteristics of the paper substrates. Page S13
Table S4. Preconcentration efficiency across targets and assays. Page S1

Table S1: The sequences of the DNA and miRNA that the paper-based device was applied for the preconcentration of the short and long dsDNA sequences and the miRNA-21 target. ds: double-stranded, MB: methylene blue.

**Name**		**Sequence (5' → 3')**
		**Colorimetric assay**
Short dsDNA	strand1	CGC**ATATATATAT**CGC
	strand2	GCGATATATATATGCG
Long dsDNA	strand1	CTAAAGACCATTGCACTTCGTGCCCGAAACGCCGA**ATATA**ATCCCAAGCGGTT TGCTGCGGTAATCATGAGGATAAGAGAGCCACGAACC
	strand2	GGTTCGTGGCTCTCTTATCCTCATGATTACCGCAGCAAACCGCTTGGGATTATA TTCGGCGTTTCGGGCACGAAGTGCAATGGTCTTTAG
		**Electrochemical assay**
MiRNA-21		uag cuu auc aga cug aug uug a
Anti-miRNA-21		Thiol-C6-TCA ACA TCA GTC TGA TAA GCT A-Atto-MB

Integration of the paper-based preconcentration device with a screen-printed electrochemical sensor

The 3D paper-based origami device consisted of 10 layers: nine 3 mm diameter discs and a tenth 9 mm diameter disc. Wax printing was used to create hydrophobic areas on each layer, enabling precise liquid handling and minimizing losses. A screen-printed electrode (SPE) was fabricated on the tenth layer, which also served as the site for analyte preconcentration, achieving the dual purpose of accumulation and measurement. The general preconcentration procedure is described in the main manuscript. Briefly, 10 µL of analyte were deposited on each layer and allowed to dry at room temperature (Fig.S1A). The device was then folded into an origami structure. Preconcentration was initiated by adding distilled water to the first layer; after 30 seconds, the device was unfolded and dried. The analyte accumulated in the bottom layer, integrated with the SPE, which was then trimmed and measured electrochemically (Fig.S1B). This design allows the combination of paper-based preconcentration with direct electrochemical detection in a single, compact platform.

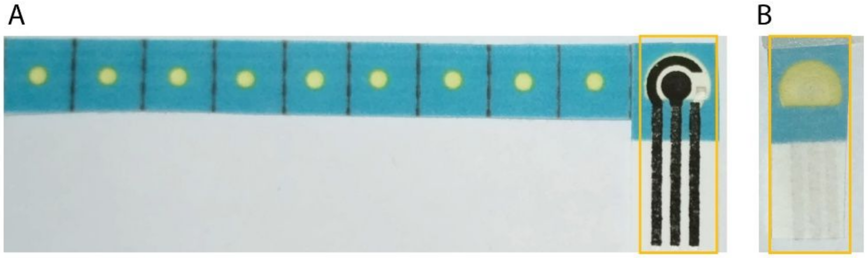

Figure S1:Integration of the paper-based preconcentration device with a screen-printed electrode (SPE). (A) The target analyte is preconcentrated in every layer of the 3D origami paper-based device. In the final layer (10th), the SPE is fabricated using a screen-printing method. (B) After analyte preconcentration, the device is unfolded, and the final layer containing the preconcentrated analyte on the SPE is cut to enable the electrochemical measurements. In these images pottasium ferricyanyde was used for the visual representaiton of the preconcetration procedure.

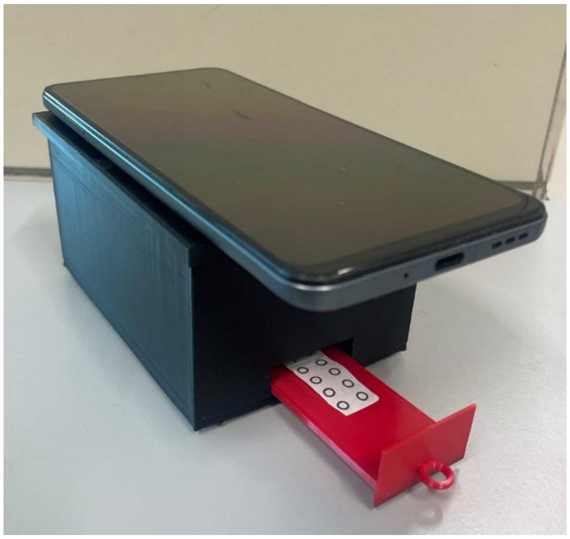

Figure S2: Assay configuration of the colorimetric nucleic acid detection. The in-house 3D-printed portable dark box was integrated with a sliding mechanism to allow easy insertion of the samples into the chamber.

Preconcentration mechanism

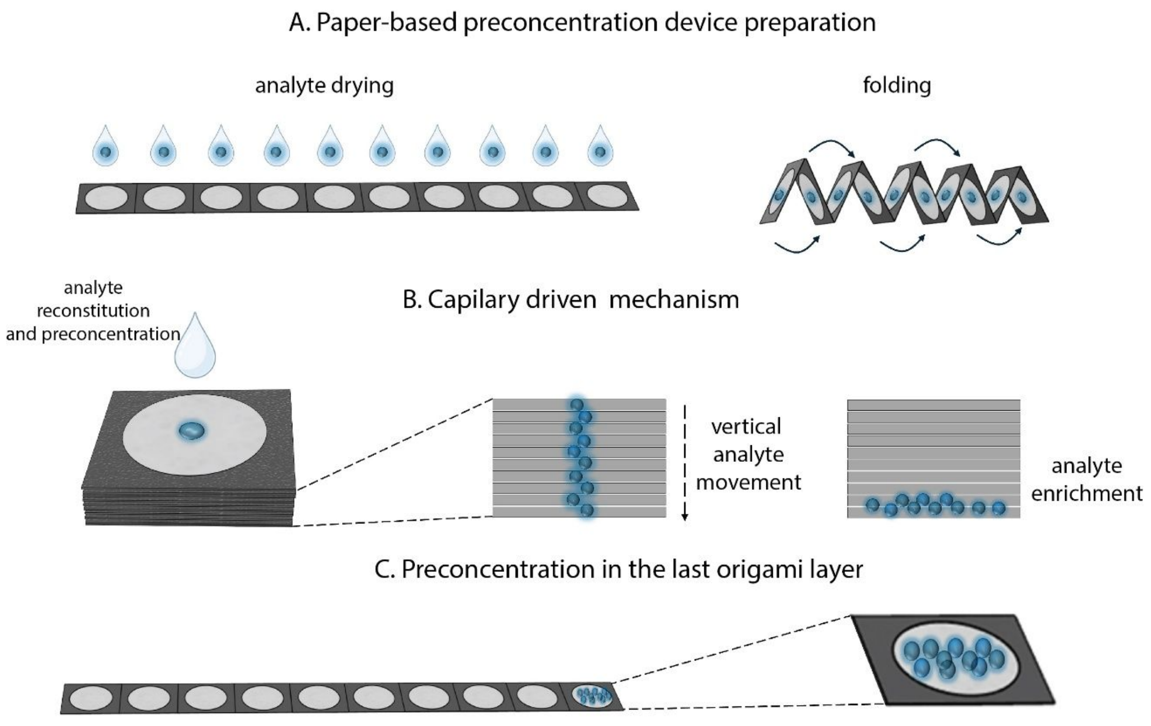

Figure S3: Schematic representation of the capillary driven mechanism of the paper-based preconcentration device.

Preconcentration evaluation of the paper-based substrates.

To evaluate the preconcentration efficiency of candidate paper-based substrates, we employed an electrochemical approach using potassium ferricyanide as a model analyte. The optimized origami configuration, as previously described, was used to assess the performance of three different substrates: 1) Whatman Grade 1 filter paper (WF1), 2) Whatman Grade 4 filter paper (WF4), and 3) commercial filter paper (CFP). For the evaluation, 10 µL of 10 mM potassium ferricyanide solution was applied to the unfolded paper-based preconcentration device and allowed to dry. After folding the origami device, the preconcentrated analyte was eluted from the final layer by adding 10 µL of water. Cyclic voltammetry (CV) was performed to determine the analyte concentration before and after preconcentration.

Measurements were conducted at a scan rate of 0.05 V/s. As illustrated in Fig.S4B for WF1, Fig.S5B for WF4 and Fig.S6B for CFP, the histograms present the current densities of the non-preconcentrated analyte (blue) and the current responses of the preconcentrated (red) analyte obtained from CV measurements for each substrate.

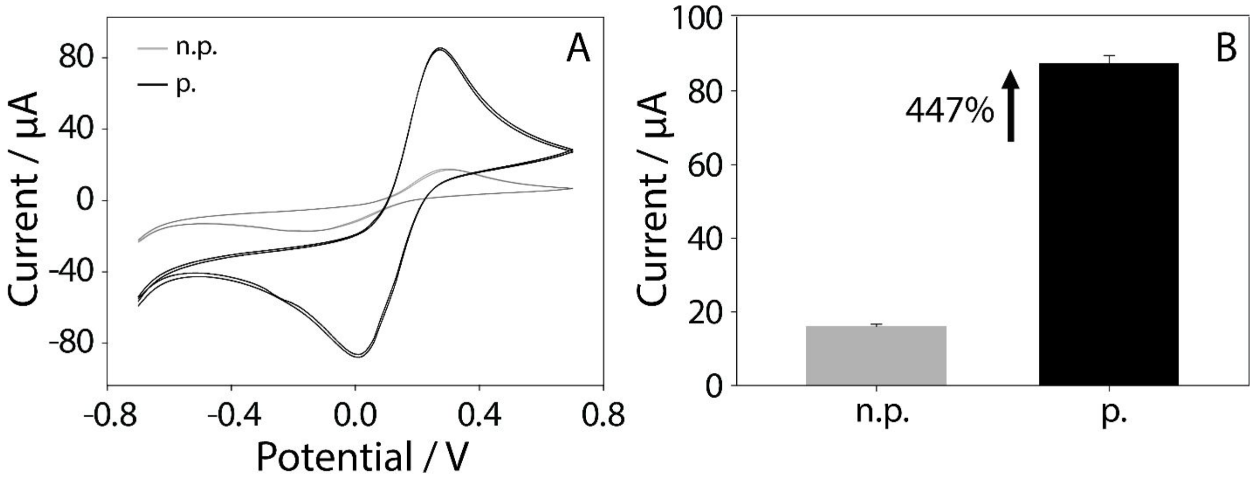

Figure S4: A) CV voltammograms of the non-preconcentrated (blue line) and the preconcentrated analyte (red line) using the WF1. B) The average current responses of the electrochemical sensor of the non-preconcentrated analyte (blue) and the preconcentrated analyte (red). Measurements were performed in six replicates. n.p.: non preconcentrated; p: preconcentrated

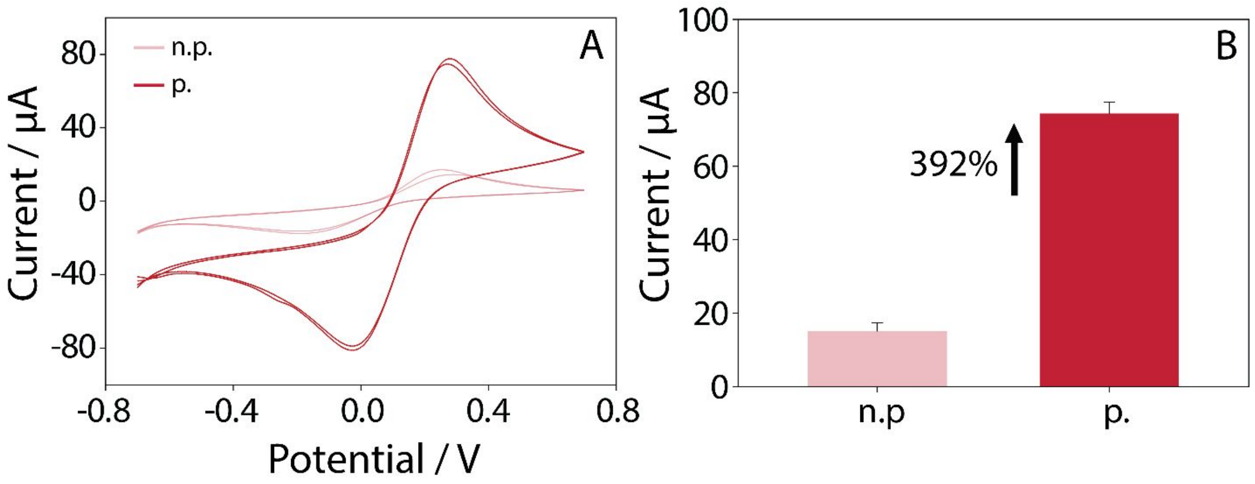

Figure S5: A) CV voltammograms of the non-preconcentrated (blue line) and the preconcentrated analyte (red line) using the WF4. B) The average current responses of the electrochemical sensor of the non-preconcentrated analyte (blue) and the preconcentrated analyte (red). Measurements were performed in six replicates. n.p.: non preconcentrated; p: preconcentrated

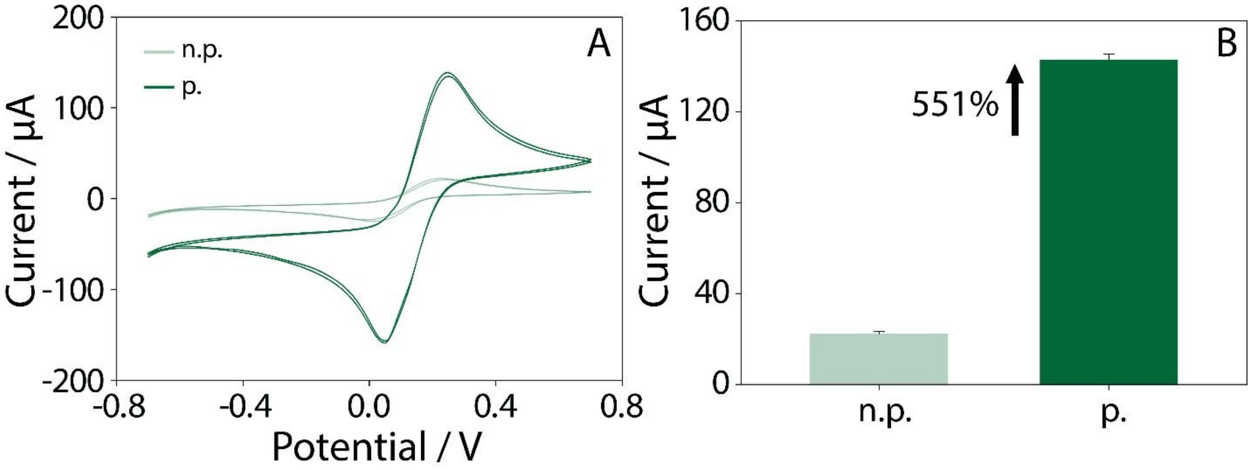

Figure S6: A) CV voltammograms of the non-preconcentrated (blue line) and the preconcentrated analyte (red line) using the CFP. B) The average current responses of the electrochemical sensor of the non-preconcentrated analyte (blue) and the preconcentrated analyte (red). Measurements were performed in six replicates. n.p.: non preconcentrated; p: preconcentrated

Dye migration experiments

Dye migration experiments were performed to further evaluate the effect of wicking and migration speed for the three paper-based substrates under investigation. Below are presented the origami paper-based devices in three strip lengths of 5, 10 and 20 layers. The various preconcentration device strips were evaluated also in different time intervals: 20 seconds, 30 seconds, 40 seconds, 1 minute and 2 minutes. This experiment was carried out for all paper substrates under investigation: WF1 (Fig. S7), WF4 (Fig. S8), and CFP (Fig. S9).

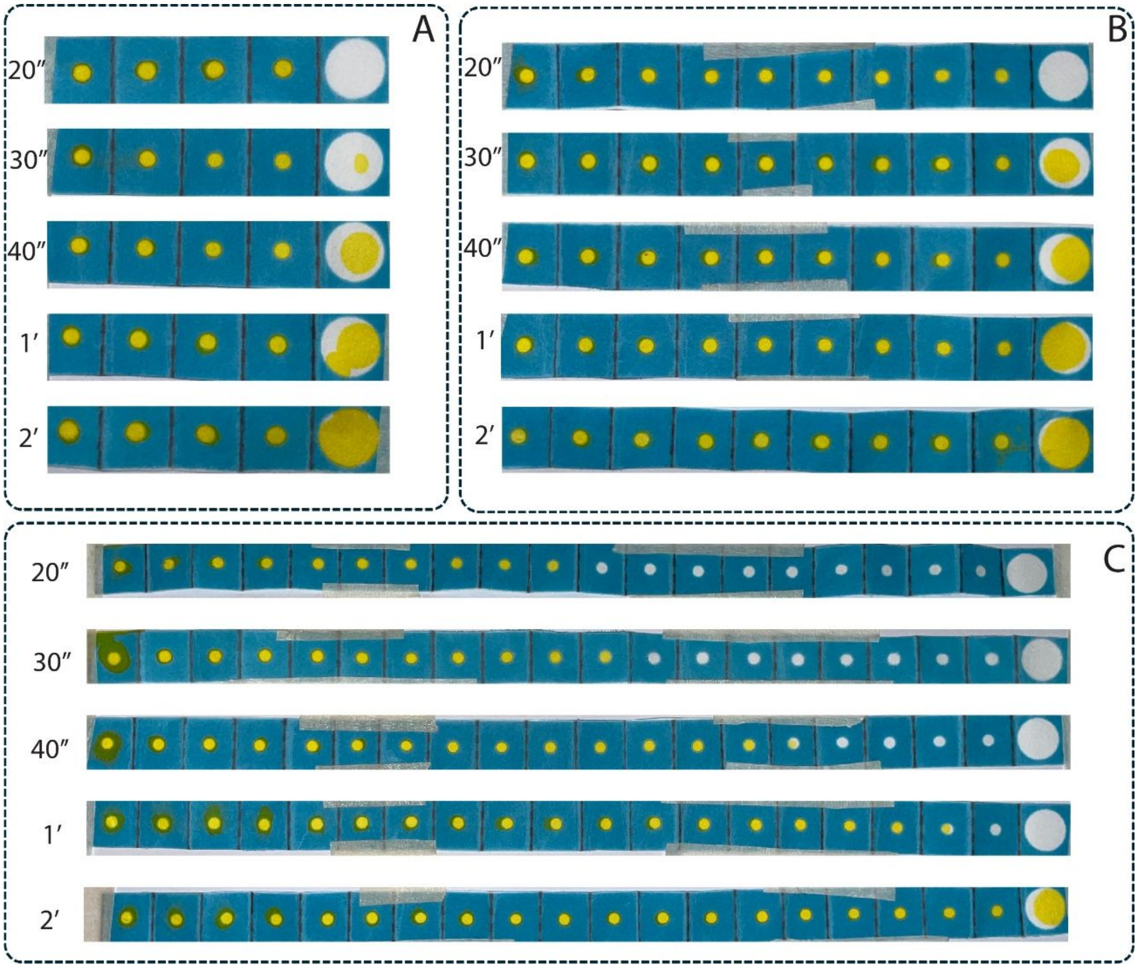

Figure S7: Dye mitigation studies for the WF1 paper substrate. The preconcentration device was designed in A) 5 layers, B) 10 layers, C) 20 layers in various unfolding time intervals namely 20 seconds, 30 seconds, 40 seconds, 1 minute and 2 minutes.

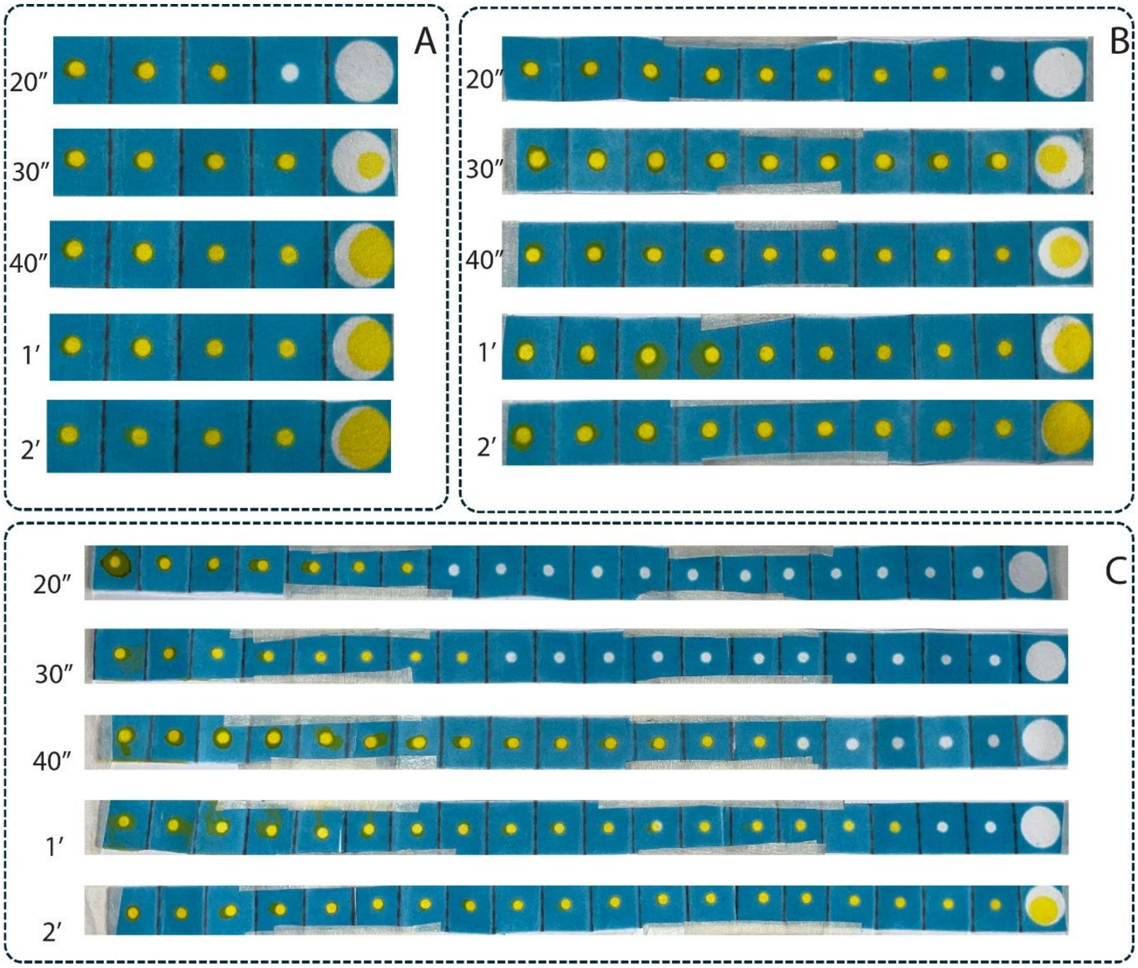

Figure S8: Dye mitigation studies for the WF4 paper substrate. The preconcentration device was designed in A) 5 layers, B) 10 layers, C) 20 layers in various unfolding time intervals namely 20 seconds, 30 seconds, 40 seconds, 1 minute and 2 minutes.

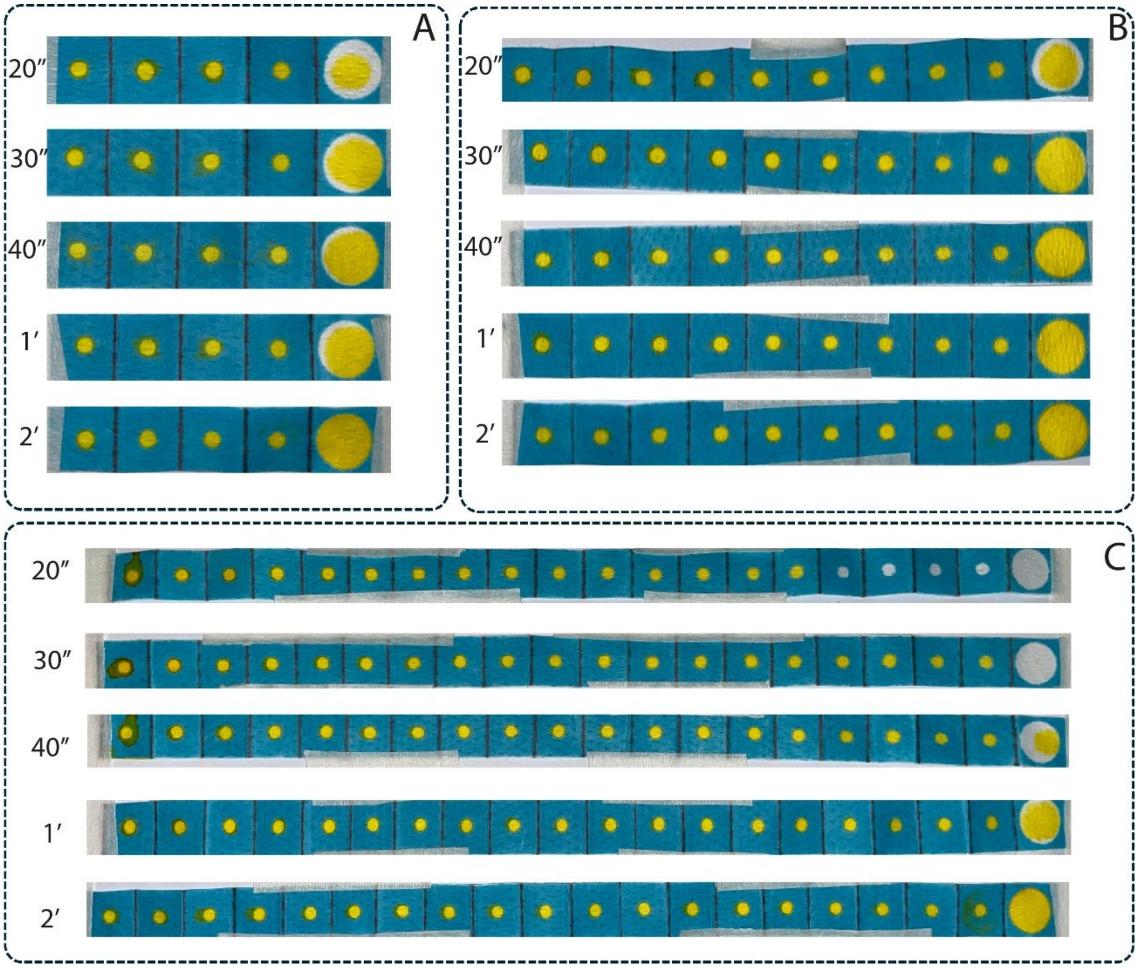

Figure S9: Dye mitigation studies for the CFP paper substrate. The preconcentration device was designed in A) 5 layers, B) 10 layers, C) 20 layers in various unfolding time intervals namely 20 seconds, 30 seconds, 40 seconds, 1 minute and 2 minutes.

Analyte recover efficiency

Figure S10 presents the three candidate paper-based substrates: WF1, WF4, and CFP. All the substrates were pretreated with wax printing (blue color) to create hydrophobic barriers for liquid handling. Each substrate is shown at three distinct stages: (i) in its dry, unused form; (ii) after the addition of 10 μL of analyte, here represented by a yellow food dye for visual tracking; (iii) after complete drying of the analyte; and (iv) after the reconstitution of the analyte with the addition of 10 μL of water. This visual sequence highlights the absorbency, spreading behavior, and retention capacity of each paper type, which are critical factors in evaluating their suitability for preconcentration applications, particularly when considered alongside their morphological and electrochemical performance assessments.

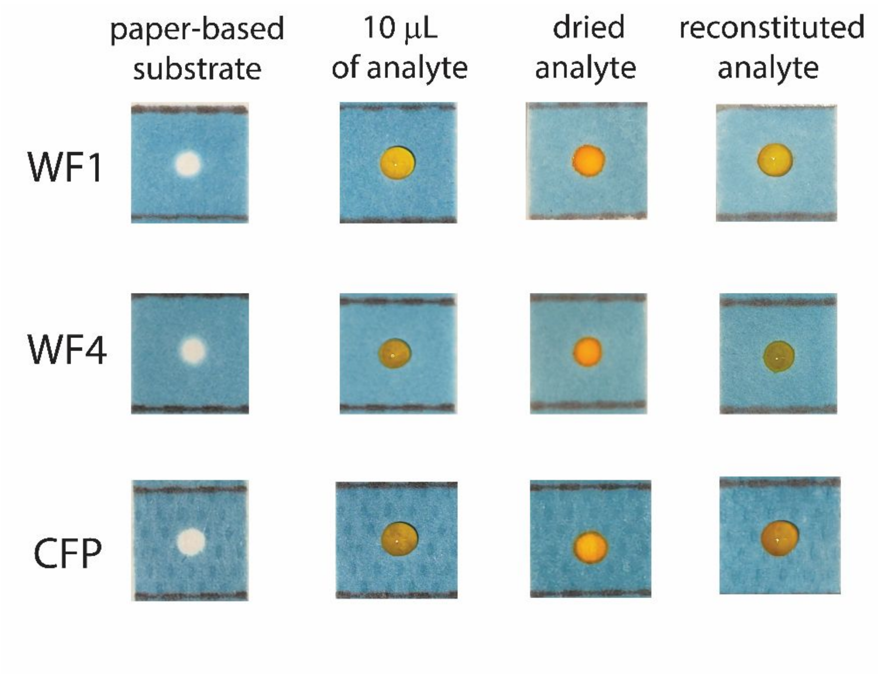

Figure S10: Visual comparison of the three paper-based substrates: WF1, WF4, and CFP. Each substrate is shown in three conditions: unused dry (left), after the addition of 10 μL of yellow food dye as a model analyte, after drying, and after the reconstitution with 10 μL of water (right). The images highlight differences in absorption, spreading, and drying behavior among the substrates.

This rehydration experiment was conducted to evaluate the recovery efficiency of each paper substrate. Briefly in this test, 10 μL of yellow food dye was first dried on the surface of each substrate. Subsequently, a 10 μL drop of water was applied directly onto the dried dye, allowing passive rehydration to occur without any mechanical agitation. The water droplet was left in contact with the substrate for various time intervals: 30 seconds, 1 minute, 2 minutes, 5 minutes, and 10 minutes. After 10 minutes, only the CFP exhibited near-complete reconstitution of the dried dye. This result indicates better interaction between the water droplet and the fiber structure of the CFP substrate, leading to a higher rehydration efficiency. In contrast, both WF1 and WF4 showed less effective dye recovery within the same time frame. Following rehydration, the recovered dye from each substrate was diluted to a final volume of 500 μL and analyzed using UV-vis spectrophotometry. The results confirmed superior analyte recovery for the CFP substrate. The corresponding absorbance spectra for each paper type at the various rehydration time points are presented in Fig. S11.

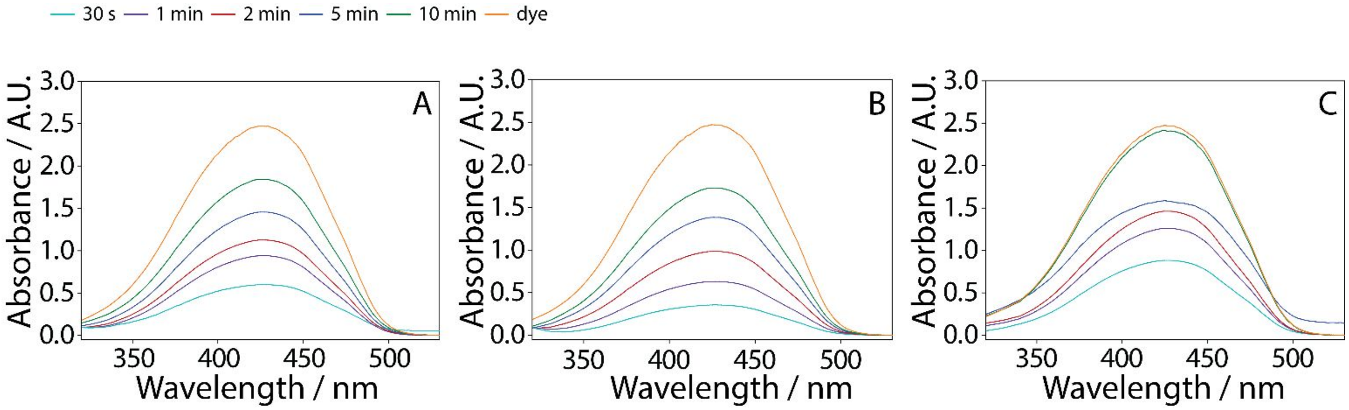

Figure S11: Rehydration experiment for all paper-based substrates: A) WF1, B) WF4, and C) CFP, evaluated at time intervals of 30 seconds (s), 1 minute (min), 2 minutes, 5 minutes, and 10 minutes for comparison with the dye solution.

Analyte mass transfer studies

To further investigate the analyte migration mechanism within the origami device potassium ferricyanide was employed as a model compound. The optimal substrate, commercial filter paper (CFP), was selected based on its superior performance and used for analyte migration studies. A 10 µL aliquot of potassium ferricyanide solution (1–100 mM) prepared in 0.1 M KCl was deposited onto a 3 mm layer of the CFP-based origami device and allowed to dry completely at room temperature. After drying the analyte was reconstituted in 50 µL of deionized water. A calibration curve was obtained from dried and reconstituted potassium ferricyanide solutions (1, 2.5, 5, 10, 20, 50, 100 mM) prepared in 0.1 M KCl (Figure S12A).

For the preconcentration step, 10 µL of 10 mM solution of potassium ferricyanide was dried on every layer of the origami device and let to dry in room temperature. After drying the device was folded as described previously and 10 µL of deionized water was added to the top layer of the assembled device allowing analyte migration through the structure for 30 seconds. The device was then unfolded and dried at room temperature. Each layer was subsequently trimmed and reconstituted in 50 µL of deionized water. The concentration of potassium ferricyanide in each layer was determined using the previously established calibration curve (Figure S12A) to quantify analyte migration and distribution across the origami layers (Figure S12B, Table S2).

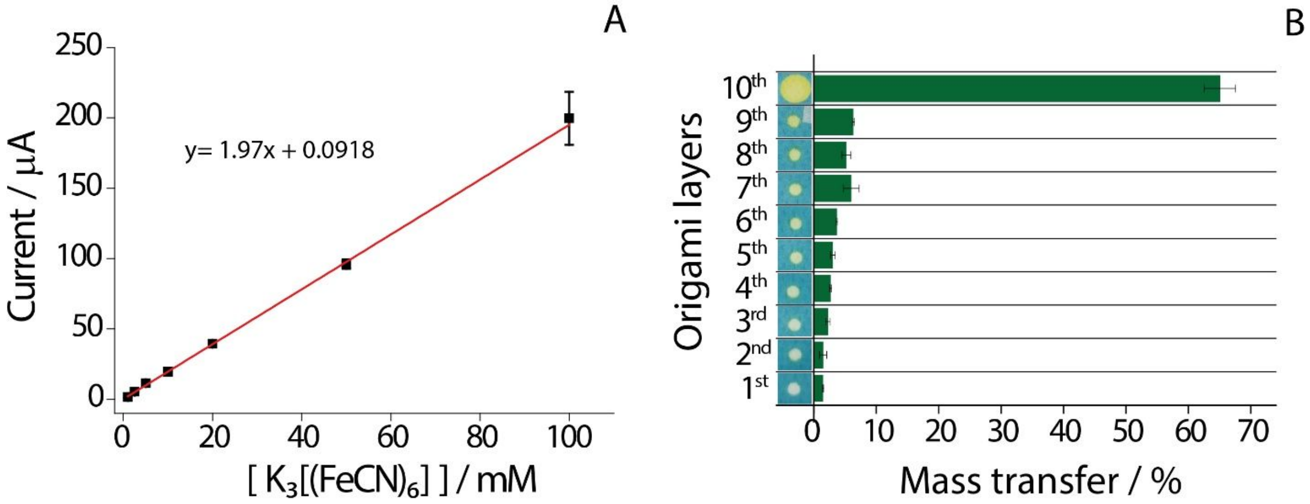

Figure S12. (A) Calibration curve obtained from dried and reconstituted potassium ferricyanide solutions, namely 1, 2.5, 5, 10, 20, 50 and 100 mM, prepared in 0.1 M KCl. (B) Mass transfer percentage of the total analyte mass across each layer of the origami device. The inset shows the corresponding color intensity for each layer, illustrating analyte accumulation. All measurements were performed in three replicates.

Table S2. Mass of analyte in each layer of the origami device following the preconcentration step. The table presents the absolute mass of analyte (µg) recovered from each layer, the corresponding percentage (%) of the total analyte mass, as well as the total analyte mass recovered from the device and its overall recovery percentage.

Origami Layer	Mass of analyte (µg)	Percentage of total analyte mass (%)
**1st**	4.87 ±0.21	1.48
**2nd**	4.93±1.90	1.50
**3rd**	7.47±0.97	2.27
**4th**	8.80±0.51	2.67
**5th**	10.00±1.19	3.68
**6th**	12.10±0.26	3.04
**7th**	19.77±4.15	6.00
**8th**	17.17±2.39	5.22
**9th**	20.81±0.60	6.32
**10th**	214.08±8.23	65.02
**Total**	320.02±20.41	97.20

Table S2: Key properties and physical characteristics of the paper substrates (WF1, WF4, and CFP) used in this study.

Properties	Whatman Grade 1 (WF1)	Whatman Grade 4 (WF4)	Commercial Filter Paper (CFP)
**Material**	cellulose	cellulose	cellulose, possible additives for strength
**Wicking speed**	150 seconds / 100 mL	37 seconds / 100 mL	Not specified (highest transfer rate observed in this work)
**Thickness**	180 µm	205 µm	135 µm
**Pore Size**	11 µm	25 µm	Not specified
**Basis weight**	87 g/m^2	92 g/m^2	67 g/m^2
**Fiber Width Mean**	13.69 µm	14.39 µm	23.20 µm
**Tensile Strength**	Moderate	Higher than Grade 1	Not specified
**Typical Applications**	General laboratory filtration, qualitative analysis	Faster filtration required, coarse particles filtration	Bench protection, sample preconcentration, general lab use
**Notable Features**	Medium flow, widely used for routine filtration	Fast filtration speed, larger pore size	Highest transfer rate and preconcentration efficiency

Preconcentration efficiency across targets and assays

The values were calculated using the calibration curve equations specific to each assay and to each target/matrix. In all cases, blank samples were also preconcentrated and taken into account when calculating the final signal enhancement. For the colorimetric assay, phosphate buffer was used as the blank, while for the electrochemical assay, phosphate-buffered saline (PBS) and human serum were used. These blank signals were subtracted from the final sample signals to ensure accurate representation of preconcentration efficiency.

Table S3: The calculated concentration of the preconcentrated samples across the different target analytes and the different colorimetric assay. ds: double stranded; Precon: preconcentration. The preconcentration experiments were performed in triplicate.

Target	Concentration before sample precon.	Calculate concentration of precon. sample	Precon. factor
**Colorimetric assay**			
short dsDNA (16 base pairs)	1 µM	6.7 µM	6.7
	5 µM	13.5 µM	2.7
	10 µM	18 µM	1.8
long dsDNA	0.25 µM	1.9 µM	8

(90 base pairs)	0.5 μM	4.3 μM	8.5
	2.5 μM	15.4 μM	2.8
**Electrochemical assay**			
miR-21 in serum	0.1 nM	1.6 nM	16
	1 nM	17.4 nM	17.4
	10 nM	180.5 nM	18
**Spectrophotometric assay**			
bovine serum albumin (66.5 kDa)	0.01 mg/mL	0.02 mg/mL	2
	0.025 mg/mL	0.036 mg/mL	1.4
	0.050 mg/mL	0.102 mg/mL	2
